# Predictive factors for the mortality of acute pancreatitis on admission

**DOI:** 10.1371/journal.pone.0221468

**Published:** 2019-08-22

**Authors:** Naruomi Jinno, Yasuki Hori, Itaru Naitoh, Katsuyuki Miyabe, Michihiro Yoshida, Makoto Natsume, Akihisa Kato, Go Asano, Hitoshi Sano, Kazuki Hayashi

**Affiliations:** 1 Department of Gastroenterology and Metabolism, Nagoya City University Graduate School of Medical Sciences, Nagoya, Japan; 2 Department of Gastroenterology, Toyokawa City Hospital, Toyokawa, Japan; University of Notre Dame Australia, AUSTRALIA

## Abstract

**Background and aims:**

The revised Atlanta classification is widely used for the evaluation of acute pancreatitis (AP) severity. However, this classification cannot be used within 48 hours of AP onset. The aim of this study was to investigate the predictive factors of mortality in patients with AP on admission.

**Methods:**

We evaluated the association between AP mortality and clinical parameters at the time of admission in patients with AP from April 2013 to December 2017 at one university hospital and one tertiary care referral center.

**Results:**

A total of 203 consecutive patients were enrolled. Nine patients (4.4%) died despite multidisciplinary treatment. In a multivariable analysis, hematocrit ≥ 40% (odds ratio [OR], 1.07; 95% confidence interval [CI], 1.01–1.13; *P* = 0.021), blood urea nitrogen (BUN) ≥ 40 mg/dL (OR, 1.26; 95% CI, 1.11–1.42; *P* < 0.001), base excess < -3.0 mmol/L (OR, 1.15; 95% CI, 1.04–1.26; *P* = 0.004), and inflammation extending to the rectovesical excavation (OR, 1.19; 95% CI, 1.10–1.30; *P* < 0.001) on admission were significantly associated with mortality.

**Conclusion:**

Among the imaging findings, inflammation extending to the rectovesical excavation was the only independent predictive factor for mortality in AP. This simple finding, obtained on computed tomography without contrast agent on admission, might be a promising prognostic factor for AP.

## Introduction

Acute pancreatitis (AP) is a common but heterogeneous pancreatic disease, ranging from mild disease to disease associated with high morbidity and mortality. Despite recent advances in diagnostic and evidence-based therapeutic management, almost 20% of patients with AP develop a complicated clinical course that requires long hospitalization, intensive care, and invasive interventions; furthermore, the condition can result in mortality [1]. The 1992 Atlanta classification [2] was one of the first attempts at grading AP. This classification divides AP into mild and severe groups. The severe group is defined by the presence of organ failure (OF) as well as local and systemic complications. Recently, two new classification systems have been developed: the 2012 revised Atlanta classification [3] and the determinant-based classification [4]. These two scales introduce the concept of persistent OF as a major determinant for the prognosis of AP. However, based on these classifications, disease severity is determined after 48 hours of admission, but AP patients sometimes die within this timeframe. Therefore, the factors that predict mortality should be further explored to provide timely treatment for AP. The aim of this study was to investigate the predictive factors of mortality in patients with AP on admission.

## Materials and methods

### Study cohort and design

**Fig 1:**
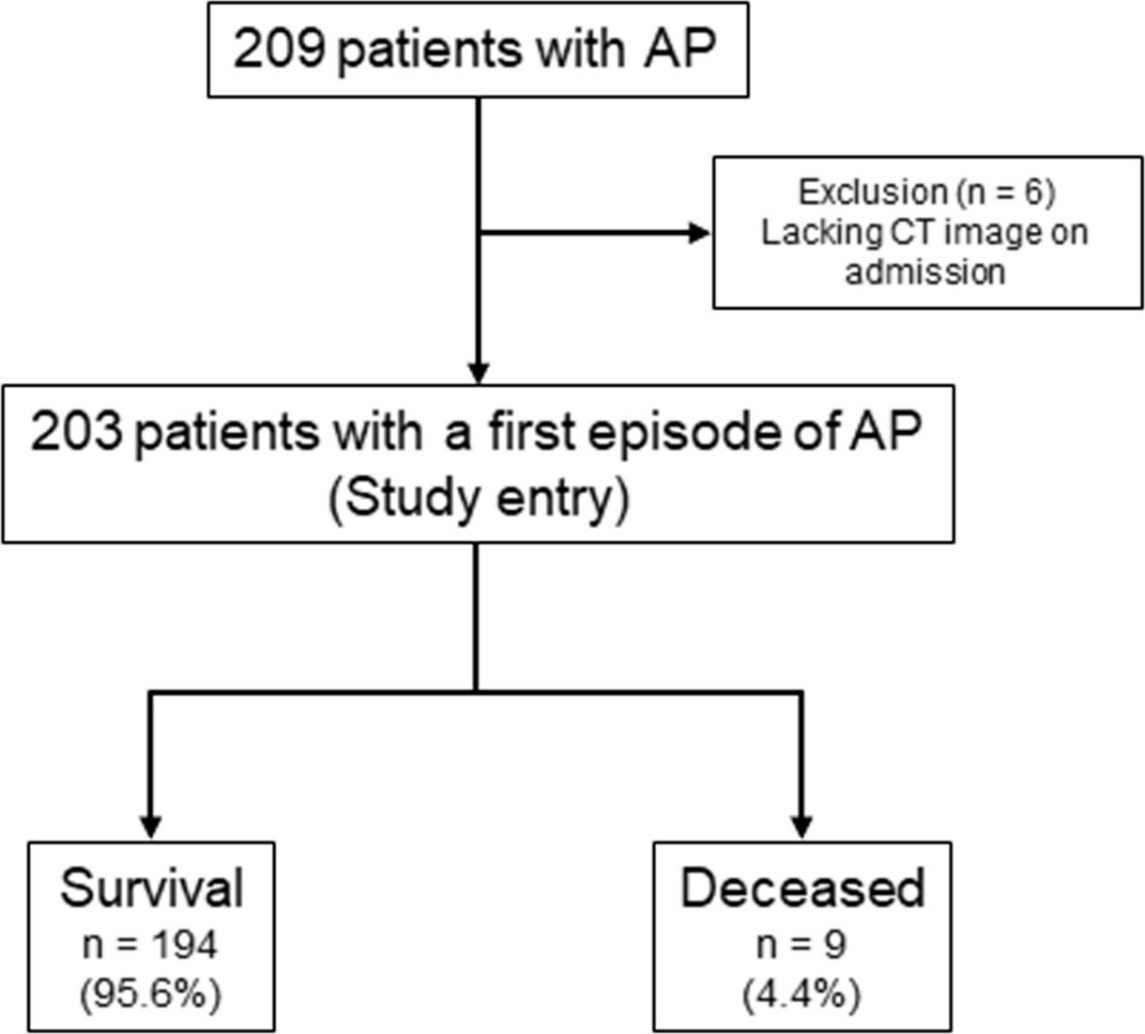
Flow diagram of patients with acute pancreatitis. *AP*, acute pancreatitis; *CT*, computed tomography.

We retrospectively evaluated 209 consecutive patients with a first episode of AP at Nagoya City University Graduate School of Medical Sciences and Toyokawa City Hospital from April 1, 2013 to December 31, 2017. No participants were deviated from the study protocol. Patients with a first episode of AP were included in this study. A diagnosis of AP was made if at least two of the following criteria were present: (1) abdominal pain compatible with AP, (2) elevation of serum lipase or amylase > 3-fold the upper limit of the normal range, and (3) computed tomography (CT) findings suggestive of AP [5]. We excluded patients who did not receive a CT on admission. Flow diagram of this study was shown in Fig 1. The follow-up period of this study ended on August 15, 2018. Patients were followed from admission to discharge. This study was approved by the Review Board of the Nagoya City University Graduate School of Medical Sciences (approval no. 60-18-0057). Actual data (S1 Fig), study protocol (S2 Fig) and opt-out protocol published on the homepage (S3 Fig) of this study were attached as supporting information files. Clinical trial registration number: UMIN000033764

### Data analysis and evaluation

**Fig 2:**
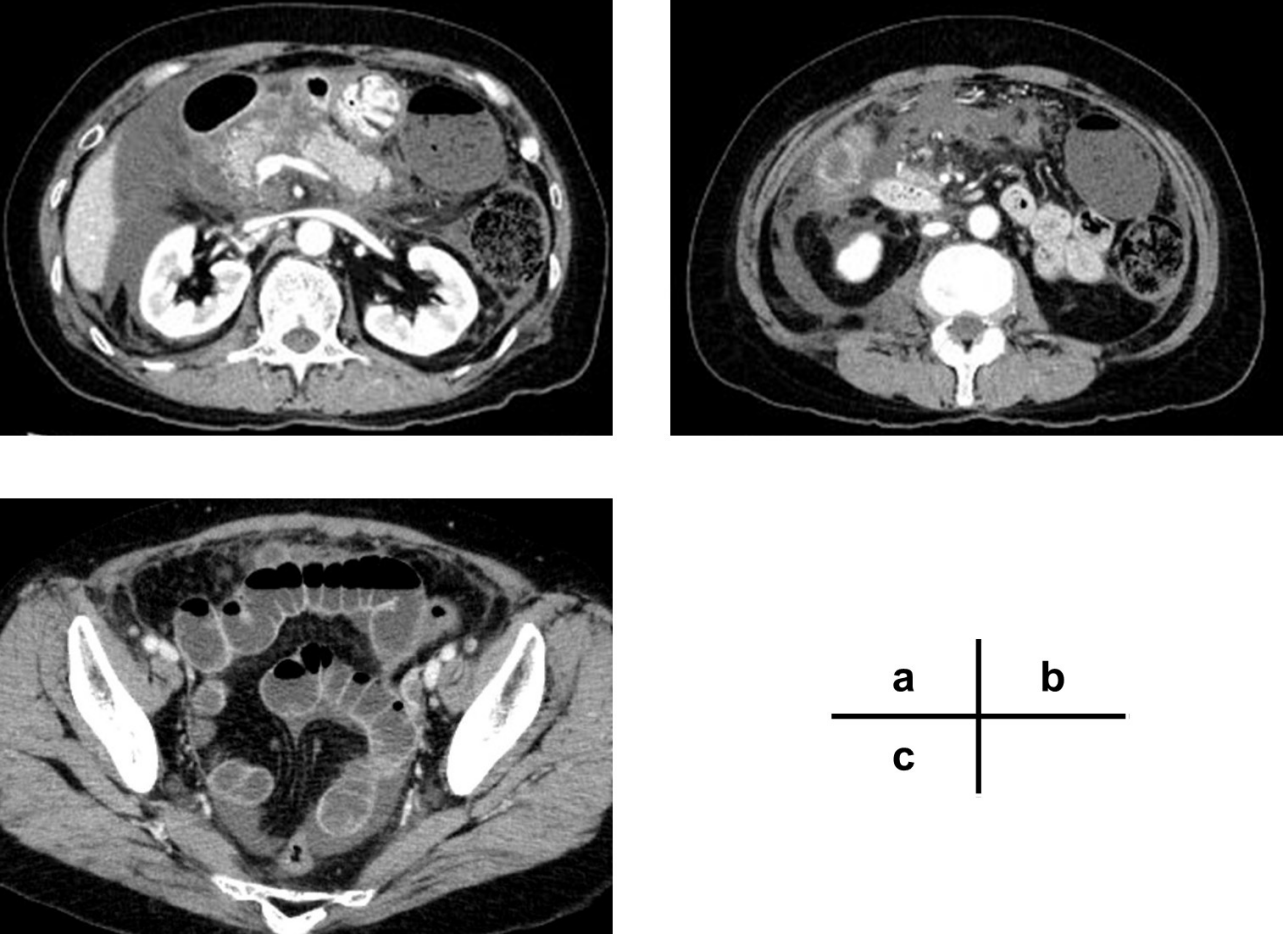
Radiologic findings of inflammation extending to the rectovesical excavation. (a) Hypo-enhanced lesion in the body of the pancreas and an ill-defined, single peripancreatic fluid collection (Balthazar score [9] D). (b) Inflammation extending over the inferior pole of the kidney. (c) Inflammation extending to the rectovesical excavation.

Records of patients with the following information were collected during the first episode of AP on admission: (1) age, (2) sex, (3) body mass index (BMI), (4) the etiology of AP, (5) time to hospital visit from initial symptom onset, (6) comorbidities, (7) vital signs (body temperature, pulse rate, and systolic blood pressure [SBP]), (8) hematology findings (white blood cell [WBC], hematocrit, platelets, C-reactive protein [CRP], amylase, lactate dehydrogenase [LDH], blood urea nitrogen [BUN], creatinine, calcium, PaO_2_, PaCO_2_, and base excess), and (9) CT findings (hypo-enhanced lesions in the pancreas, acute peripancreatic fluid collection, inflammation extending to the inferior pole of the kidney/rectovesical excavation). Acute Physiology and Chronic Health Evaluation (APACHE) II scores [6], the Charlson comorbidity index [7], and systemic inflammatory response syndrome (SIRS) scores [8] were also individually assessed for each patient. We used the Revised Atlanta classification [3] and the determinant-based classification [4] systems to assess AP severity. Typical image findings were shown in Fig 2; (a) hypo-enhanced lesion in the body of the pancreas and an ill-defined, single peripancreatic fluid collection, (b) inflammation extending over the inferior pole of the kidney, and (c) inflammation extending to the rectovesical excavation.

Treatment of our study included: (i) enteral feeding, (ii) the amount of infused volume within the first 24 hours, (iii) continuous hemodiafiltration, (iv) requirement for intensive care unit (ICU) treatment and length of stay, (v) endoscopic sphincterotomy, (vi) endoscopic intervention, and (vii) surgical intervention. Endoscopic interventions included endoscopic drainage and/or necrosectomy for infected acute necrotic collection or walled-off necrosis (WON). Surgical interventions were defined as percutaneous drainage, open/laparoscopic debridement, and/or drainage with or without pancreatic resection. Diagnosis of WON was made according to the 2012 revised Atlanta classification [3]; consisted of necrotic tissue contained within an enhancing wall of reactive tissue that occurred ≥ 4 weeks after onset of AP. The management and treatment strategy of WON was followed to the step-up approach [10]. It is proposed as an alternative to open necrosectomy. Less invasive techniques, including percutaneous/endoscopic drainage and retroperitoneal necrosectomy are being used increasingly which aimed to control infection, rather than complete removal of the infected necrotic tissue.

Clinical outcomes during hospitalization were evaluated according to the following criteria: mortality, urine volume within the first 24 hours, transient/persistent OF, WON, and the length of hospital stay. OF was defined by shock (SBP < 90 mmHg), pulmonary insufficiency (PaO_2_ < 60 mmHg on room air or mechanical ventilation requirement), or renal failure (serum creatinine level > 2 mg/dL or need for hemodialysis). Persistent OF was defined as OF lasting for over 48 hours.

We compared baseline characteristics, physical and blood exams, and CT findings on admission between AP patients who died and those who survived at discharge. Each parameter described above was evaluated as a predictive factor for mortality in univariable and multivariable analyses.

### Statistical analysis

The Chi-square test and Fisher’s exact test were used to compare categorical variables. The Mann-Whitney U-test was used to compare continuous variables. A *P*-value < 0.05 was considered to indicate statistical significance, and statistical tests were two-sided. Factors with *P* < 0.2 in univariable analyses were considered potential risk factors and were included in the multivariable analysis. Logistic regression models were used in multivariable analyses. We excluded hypo-enhanced lesions in the pancreas in the multivariable analysis on the grounds that several cases lacked contrasted-enhanced CT (CECT) on admission. All statistical analyses were performed using the SPSS software (version 23; IBM Corporation, USA).

## Results

### Study flow and patient characteristics on admission

Of the 209 patients with AP, six lacked CT images on admission; therefore, 203 patients were enrolled in this study. Nine patients (4.4%) died despite multidisciplinary treatment (Fig 1). The demographic and clinical characteristics of the patients are shown in Table 1. There was a slight male predominance in our population (58.6%), with a mean age of 65.0 ± 18.3 years. The most common two etiologies of AP were biliary (n = 75, 36.9%) and idiopathic (n = 65, 32.0%). A total of 111 cases (54.7%) underwent CECT on admission, whereas 143 cases (68.4%) underwent CECT 48 hours after admission. Among the cases who received CECT on admission, 16.2% (18/111) had hypo-enhanced lesions in the pancreas. Acute peripancreatic fluid collection and inflammation extending to the inferior pole of the kidney and the rectovesical excavation on CT were found in 22.7% (46/203), 16.7% (34/203) and 10.8% (22/203) of patients, respectively.

**Table 1:** Patient baseline characteristics on admission (n = 203).

Baseline characteristics	
Age, year, mean ± SD	65.0 ± 18.3
Sex, male/female	119/84
BMI, kg/m^2^, mean ± SD	22.8 ± 4.2
Etiology	
Alcohol	45 (22.2)
Biliary	75 (36.9)
Idiopathic	65 (32.0)
Tumor	3 (1.5)
Anastomotic stricture	7 (3.4)
Drug	3 (1.5)
Other	5 (2.5)
Time to hospital visit from initial symptom onset, hour, mean ± SD	23.9 ± 35.1
Charlson comorbidity index, score, mean ± SD	0.9 ± 1.5
APACHE II, score, mean ± SD	8.3 ± 4.3
SIRS, score, mean ± SD	1.0 ± 0.9
Physical and blood exam	
Body temperature, degree, mean ± SD	36.9 ± 0.8
Pulse rate, /minute, mean ± SD	78.9 ± 16.6
SBP, mmHg, mean ± SD	134.8 ± 27.5
WBC, × 10^3^/μL, mean ± SD	11.2 ± 4.6
Platelet, × 10^4^/μL, mean ± SD	21.8 ± 7.1
CRP, mg/dL, mean ± SD	3.6 ± 6.5
Amylase, U/L, mean ± SD	1400.0 ± 1270.8
LDH, U/L, mean ± SD	333.8 ± 195.6
BUN, mg/dL, mean ± SD	19.4 ± 12.7
Creatinine, mg/dL, mean ± SD	0.9 ± 0.6
Calcium, mg/dL, mean ± SD	9.1 ± 0.6
PaO_2_, mmHg, mean ± SD	79.9 ± 15.8
PaCO_2_, mmHg, mean ± SD	38.4 ± 7.0
Base excess, mmol/L, mean ± SD	0.1 ± 3.3
CT findings	
Hypo-enhanced lesions in the pancreas	18/111^†^ (16.2)
Acute peripancreatic fluid collection	46 (22.7)
Inflammation extending to the inferior pole of kidney	34 (16.7)
rectovesical excavation	22 (10.8)

Values are presented as number of cases with percents in parentheses, unless otherwise indicated.

APACHE, Acute Physiology and Chronic Health Evaluation; BMI, body mass index; BUN, blood urea nitrogen; CRP, C-reactive protein; CT, computed tomography; LDH, lactate dehydrogenase; SBP, systolic blood pressure; SD, standard deviation; SIRS, systemic inflammatory response syndrome; WBC, white blood cell.

^†^, A total of 111 cases underwent contrasted-enhanced CT on admission.

### Severity, treatment, and clinical outcomes

As shown in Table 2, according to the revised Atlanta classification [3], 45.8%, 44.8%, and 9.4% of patients were defined as having mild, moderate, and severe AP, respectively. Using the determinant-based classification [4], 73.4%, 11.8%, 13.3%, and 1.5% of cases were defined as mild, moderate, severe, and critical, respectively. Fifty-three cases (26.1%) underwent enteral feeding with a nasoenteric tube. A total of 10 cases (4.9%) required admission to the ICU, with mean duration of 7.7 ± 3.7 days. Endoscopic sphincterotomy was conducted in 56 patients (27.6%) for the treatment of biliary pancreatitis. Endoscopic and surgical intervention was performed for WON in two (1.0%) cases and one (0.5%) case, respectively. Overall, nine patients (4.4%) died. Transient and persistent OF was observed in 12.3% (n = 25) and 9.4% (n = 19) of cases, respectively. Patients all who died were due to complications which related to pancreatitis. The causes of death were multiple OF in seven patients, peritonitis due to stomach perforation from WON in one, and gastrointestinal bleeding from newborn blood vessel in one, respectively.

**Table 2:** Severity, treatment and clinical outcomes (n = 203).

Severity	
Revised Atlanta classification	
Mild	93 (45.8)
Moderately severe	91 (44.8)
Severe	19 (9.4)
The determinant-based classification of acute pancreatitis severity	
Mild	149 (73.4)
Moderate	24 (11.8)
Severe	27 (13.3)
Critical	3 (1.5)
Treatment	
Enteral feeding	53 (26.1)
The amount of infused volume within first 24 hours, ml, mean ± SD	3988.4 ± 1353.7
Continuous hemodiafiltration	7 (3.4)
ICU stay	10 (4.9)
duration, day, mean ± SD	7.7 ± 3.7
Endoscopic sphincterotomy	56 (27.6)
Endoscopic intervention	2 (1.0)
Surgical intervention	1 (0.5)
Clinical outcomes	
Mortality	9 (4.4)
Urine volume within the first 24 hours, ml, mean ± SD	1414.0 ± 778.2
Transient organ failure	25 (12.3)
Persistent organ failure	19 (9.4)
Walled-off necrosis	14 (6.9)
Hospital stay, day, mean ± SD	15.8 ± 14.7

Values are presented as number of cases with percents in parentheses, unless otherwise indicated.

ICU, intensive care unit; SD, standard deviation.

### Predictive factors for mortality on admission

Table 3 shows the results of the univariable analysis to determine the predictive factors for mortality. The following factors were significantly associated with mortality in the univariable analysis: idiopathic etiology (*P* = 0.023), APACHE II (*P* = 0.036), SIRS (*P* = 0.016), pulse rate (*P* = 0.032), platelet levels (*P* = 0.027), amylase levels (*P* = 0.043), creatinine levels (*P* = 0.002), hypo-enhanced lesions in the pancreas (*P* = 0.001), inflammation extending to the inferior pole of the kidney (*P* = 0.045), and inflammation extending to the rectovesical excavation (*P* < 0.001).

**Table 3:** Comparison of deceased and survival patients on admission (n = 203).

	Deceased (n = 9)	Survival (n = 194)	*P* value
Baseline characteristics			
Age, year, mean ± SD	76.6 ± 18.3	64.4 ± 18.2	0.052
Sex, male/female	3/6	116/78	0.115
BMI, kg/m^2^, mean ± SD	19.2 ± 9.1	22.8 ± 4.5	0.272
Etiology			
Alcohol	1 (11.1)	44 (22.7)	0.414
Biliary	2 (22.2)	73 (37.6)	0.349
Idiopathic	6 (66.7)	59 (30.4)	0.023*
Tumor	0 (0.0)	3 (1.5)	> 0.999
Anastomotic stricture	0 (0.0)	7 (3.6)	> 0.999
Drug	0 (0.0)	3 (1.5)	> 0.999
Other	0 (0.0)	5 (2.6)	> 0.999
Time to hospital visit from initial symptom onset, hour, mean ± SD	16.9 ± 14.2	24.2 ± 35.9	0.545
Charlson comorbidity index, score, mean ± SD	1.8 ± 1.8	0.8 ± 1.5	0.061
APACHE II, score, mean ± SD	14.8 ± 8.0	8.1 ± 3.8	0.036*
SIRS, score, mean ± SD	1.8 ± 1.0	1.0 ± 0.9	0.016*
Physical and blood exam			
Body temperature, degree, mean ± SD	36.4 ± 0.8	36.9 ± 0.8	0.067
Pulse rate, /minute, mean ± SD	90.4 ± 23.8	78.3 ± 16.1	0.032*
SBP, mmHg, mean ± SD	125.0 ± 51.6	135.3 ± 26.1	0.569
WBC, × 10^3^/μL, mean ± SD	10.5 ± 5.8	11.2 ± 4.6	0.618
Hematocrit, %, mean ± SD	43.3 ± 5.8	40.4 ± 5.3	0.112
Platelet, × 10^4^/μL, mean ± SD	16.6 ± 8.3	22.0 ± 7.0	0.027*
CRP, mg/dL, mean ± SD	7.5 ± 11.0	3.4 ± 6.2	0.302
Amylase, U/L, mean ± SD	2828.4 ± 1859.0	1333.8 ± 1206.5	0.043*
LDH, U/L, mean ± SD	460.8 ± 386.9	329.7 ± 183.4	0.341
BUN, mg/dL, mean ± SD	42.8 ± 33.2	18.4 ± 9.9	0.058
Creatinine, mg/dL, mean ± SD	1.6 ± 0.8	0.9 ± 0.6	0.002**
Calcium, mg/dL, mean ± SD	9.6 ± 1.9	9.1 ± 0.5	0.550
PaO_2_, mmHg, mean ± SD	75.3 ± 24.4	80.2 ± 15.5	0.555
PaCO_2_, mmHg, mean ± SD	37.6 ± 7.5	38.4 ± 7.1	0.721
Base excess, mmol/L, mean ± SD	-4.6 ± 6.5	0.2 ± 2.8	0.077
CT findings			
Hypo-enhanced lesion in the pancreas, Yes/No/Unknown	2/0/7	16/93/85	0.001**
Acute peripancreatic fluid collection	0 (0.0)	46 (22.7)	0.214
Inflammation extending to the inferior pole of kidney	4 (44.4)	30 (14.8)	0.045*
rectovesical excavation	5 (55.6)	17 (8.8)	< 0.001**

Values are presented as number of cases with percents in parentheses, unless otherwise indicated.

APACHE, Acute Physiology and Chronic Health Evaluation; BMI, body mass index; BUN, blood urea nitrogen; CRP, C-reactive protein; CT, computed tomography; LDH, lactate dehydrogenase; SBP, systolic blood pressure; SD, standard deviation; SIRS, systemic inflammatory response syndrome; WBC, white blood cell.

*, *P* < 0.05

**, *P* < 0.01.

In the multivariable analysis, hematocrit ≥ 40% (odds ratio [OR], 1.07; 95% confidence interval [CI], 1.01–1.13; *P* = 0.021), BUN ≥ 40 mg/dL (OR, 1.26; 95% CI, 1.11–1.42; *P* < 0.001), base excess < -3.0 mmol/L (OR, 1.15; 95% CI, 1.04–1.26; *P* = 0.004), and inflammation extending to the rectovesical excavation (OR, 1.19; 95% CI, 1.10–1.30; *P* < 0.001) were significant factors associated with mortality (Table 4).

**Table 4:** Predictors of mortality on admission findings.

Variable	Multivariable analysis	
	OR	*P* value
Age (≥ 70)	1.04 (0.99–1.11)	0.151
Gender (Male)	0.99 (0.93–1.04)	0.627
Etiology (Idiopathic)	1.05 (1.00–1.11)	0.076
Charlson comorbidity index (≥ 1)	1.05 (0.99–1.10)	0.095
APACHE II (≥ 8)	1.00 (0.94–1.06)	0.943
SIRS (≥ 3)	0.98 (0.88–1.09)	0.701
Body temperature (≥ 37.0 degrees)	0.97 (0.92–1.02)	0.262
Pulse rate (≥ 90 /minute)	1.02 (0.95–1.08)	0.637
Hematocrit (≥ 40%)	1.07 (1.01–1.13)	0.021*
Platelet (< 10 × 10^4^/μL)	1.11 (0.96–1.27)	0.147
Amylase (≥ 1000 U/L)	1.03 (0.97–1.08)	0.333
BUN (≥ 40 mg/dL)	1.26 (1.11–1.42)	< 0.001**
Creatinine (≥ 2.0 mg/dL)	1.02 (0.89–1.15)	0.799
Base excess (< -3.0 mmol/L)	1.15 (1.04–1.26)	0.004**
Inflammation extending to the inferior pole of kidney (Yes)	1.07 (1.00–1.15)	0.063
Inflammation extending to the rectovesical excavation (Yes)	1.19 (1.10–1.30)	< 0.001**

Total number of patients, n = 203; deceased, n = 9; survival, n = 194.

APACHE, Acute Physiology and Chronic Health Evaluation; BUN, blood urea nitrogen; OR, Odds ratio (95%CI); SIRS, systemic inflammatory response syndrome.

*, *P* < 0.05

**, *P* < 0.01.

Patients who had inflammation extend to rectovesical excavation required significant higher hospitalization cost (1,410,620 vs 761,218 Japanese yen; *P* = 0.022) (Table 5).

**Table 5:** Comparison of total cost between inflammation extend to rectovesical excavation group and the other group (n = 203).

	Inflammation extend to rectovesical excavation (n = 22)	Other (n = 181)	*P* value
Total cost^†^, yen	1,410,620	761,218	0.022*

Values are presented in Japanese yen.

^†^, Each cost equals to approximately 12,834 and 6,925 US dollars, respectively.

*, *P* < 0.05

## Discussion

In the present study, hematocrit levels (≥ 40%), BUN levels (≥ 40 mg/dL), base excess (< -3.0 mmol/L), and inflammation extending to the rectovesical excavation on admission were predictive factors for mortality in AP patients in a multivariable analysis. Among the imaging findings, inflammation extending to the rectovesical excavation was the only independent predictive factor. An imaging finding that can be assessed without the use of contrast is valuable, especially in patients who are unable to tolerate contrast medium.

AP remains a heterogeneous and poorly understood disease, ranging from a mild clinical course to severe life-threatening complications [11]. Early evaluation of the severity of AP is essential to allow the clinician to predict patient outcome, estimate prognosis, and determine the need for ICU care. Recently, it has been reported that the most useful predictors for severe AP are elevated BUN and creatinine levels and an elevated hematocrit, particularly if levels do not return to the normal range after fluid resuscitation [5, 12]. Although we only evaluated data on admission, similar to previous studies, BUN and hematocrit were important indicators of disease severity and mortality in our study.

Takeda *et al*. [13] proposed a scoring system for AP that includes prognostic factors and CT grade on CECT, including extrapancreatic progression of inflammation in the anterior pararenal space (0 point), root of the mesocolon (1 point), and beyond the lower pole of the kidney (2 point). Disease severity is correlated with the extent of inflammation into internal organs away from the pancreas. This scoring system was constructed for the purpose of detecting patients with early stage severe AP who require transfer to a specialist medical center or ICU to receive adequate treatment. While this classification is useful to detect severe early stage AP, a certain number of patients who did not have severe AP based on the revised Atlanta or determinant-based classifications were defined as having severe AP. Based on clinical practice using this scoring system, the definition of severe AP (not in revised Atlanta or determinant-based classification) is based on extrapancreatic progression of inflammation. In the present study, 27.6% of the patients (56/203) were defined as having severe AP according to CT findings based on inflammation progression, and only 16.1% (9/56) of the cohort died. Therefore, we recommend adding the finding of inflammation extension to the rectovesical excavation as an important score item, as it seems adequate for predicting mortality. Furthermore, recent topic in outcome of healthcare is cost. From our present study, the finding of inflammation extension to the rectovesical excavation is also useful to predict the higher cost to treat the patients. Differentiating extrapancreatic progression of inflammation from reactive change becomes difficult in patients who have received large volumes of extracellular fluid. Thus, in this study, we used only CT findings on admission before fluid hydration was initiated. Although nearly 70% of the patients underwent CECT within 48 hours after admission, only 55% underwent CECT on admission. This is a limitation of this study, as we were unable to evaluate hypo-enhanced pancreatic lesions on admission. The reasons for not performing CECT included decreased renal function, allergy to contrast medium, and mild disease that did not warrant CECT according to the attending physician.

CECT is essential to detect necrosis. However, necrosis is not usually present on admission and may develop after 24–48 hours [9, 14]. In early stage AP, necrosis may present as hypo-enhanced pancreatic lesions. In the present study, 16.2% (18/111) of the patients had hypo-enhanced lesions in the pancreas, and of these, only 11.1% (2/18) died. Using only CECT to predict early stage pancreatic necrosis is challenging as its sensitivity ranges from 53–78%, its specificity ranges from 88–90%, and the timing of performing CT is debated [15–17]. Pancreatic necrosis is one of the triggers of persistent OF; therefore, detecting it accurately and early may predict the onset of persistent OF. In recent years, perfusion CT has been reported to be useful in predicting early stage pancreatic necrosis (sensitivity, 88–100%; specificity, 84–100%) [18–20]. However, perfusion CT requires specific equipment and it is not available at all institutions currently. This useful and crucial imaging method is expected to be more widely available in the future. Furthermore, if early stage pancreatic necrosis is detected, it is important to initiate treatment in patients to halt the progression to complete necrosis.

Our study has certain limitations. First, it was retrospective in nature and the treatments administered were based on the experience of the physicians with limited cases. Second, as mentioned above, not all patients underwent CECT on admission and, therefore, we were unable to evaluate the presence of hypo-enhanced pancreatic lesions in all patients. Third, the number of deceased patients in this study was not completely sufficient for precise multivariable analysis to figure out the predictors for the mortality of AP. To fulfill the enough number of patients for statistically sufficient multivariable analysis, nation- or world-wide study might be required. Future large-scale randomized prospective studies are warranted. However, we believe that this report would be helpful for extracting and weighting among the numerous factors those which considered to be related to the severity of AP.

In conclusion, hematocrit, BUN, base excess, and inflammation extending to the rectovesical excavation on admission were predictive factors for mortality in AP patients. Moreover, inflammation extending to the rectovesical excavation was the only independent predictive factor among the imaging findings. This simple CT finding that can be assessed without the use of contrast might be a promising prognostic factor for AP.

## Supporting information

S1 Fig Actual data of this study cohort. (XLSX)

S2 Fig Study protocol of this study. (PDF)

S3 Fig Opt-out protocol that published on the homepage. (PDF)

## Abbreviations

AP: acute pancreatitis
APACHE: Acute Physiology and Chronic Health Evaluation
AUC: area under the curve
BMI: body mass index
BUN: blood urea nitrogen
CECT: contrasted-enhanced CT
CI: confidence interval
CRP: C-reactive protein
CT: computed tomography
ICU: intensive care unit
LDH: lactate dehydrogenase
OF: organ failure
OR: odds ratio
SBP: systolic blood pressure
SIRS: systemic inflammatory response syndrome
WBC: white blood cell
WON: walled-off necrosis
