# Supplementary material for: Predictive factors for the mortality of acute pancreatitis on admission
Source: PLoS One. 2019 Aug 22;14(8):e0221468. doi: 10.1371/journal.pone.0221468 (PMC6706052; doi:10.1371/journal.pone.0221468)
Supplement: S2 Fig — (PDF) [file pone.0221468.s002.pdf]

実施計画書

研究課題名

急性膵炎の入院時における死亡予測因子の検討

作成日：第 1 版　平成 30 年 6 月 1 日

名古屋市立大学病院　消化器代謝内科学

責任医師　：　肝膵内科　内藤格

連絡先：PHS 3331

E-mail Address：inaito@med.nagoya-cu.ac.jp

1. 研究課題名

急性膵炎の入院時における死亡予測因子の検討

2. 研究の実施体制・研究組織

2. 1. 研究者氏名

研究責任者

名古屋市立大学病院　消化器代謝内科学　肝膵内科　内藤格
〒467-8601　名古屋市瑞穂区瑞穂町川澄 1
連絡先：TEL　052-853-8211　 FAX　052-852-0952　PHS 3331

研究分担者

名古屋市立大学病院　消化器代謝内科学　肝膵内科：神野成臣（大学院生），堀寧（臨床研究医），林香月　（講師），宮部勝之（助教），吉田道弘（助教），夏目まこと（大学院生），加藤晃久（臨床研究医），浅野剛（臨床研究医）

2. 2. 研究事務局

名古屋市立大学病院　消化器代謝内科学　肝膵内科　内藤格
〒467-8601　名古屋市瑞穂区瑞穂町川澄 1
連絡先：TEL　052-853-8211　 FAX　052-852-0952　PHS 3331

2. 3. 参加予定施設および各施設研究責任者

豊川市民病院　消化器内科　佐野仁

3. 本研究の背景、意義および科学的合理性の根拠

急性膵炎とは膵臓の急性炎症で，他の隣接する臓器や遠隔臓器にも影響を及ぼしえる疾患である. 発生頻度は 5～80/10 万人/年であり, 近年増加傾向にある. ガイドラインの普及などによって急性膵炎診療の標準化や早期診断/治療が行われるようになり，急性膵炎および重症急性膵炎の予後は改善傾向にある．しかしながら重症の急性膵炎はいまだ高い死亡率であり，満足すべきものではない.

感染性膵壊死に対する経皮的・内視鏡的ドレナージなどの進歩に加えて，経時的な造影 CT 所見の蓄積から，急性膵炎の形態分類の再検討が行われ，2012 年に改訂アトランタ分類が発表された [1)2)]. 膵局所合併症の定義も大幅に改定され，改訂アトランタ群類は現在世界的に最も標準的な急性膵炎の重症度分類として利用されている．しかしながら改訂アトランタ分類は Modified Marshall score を用いて臓器不全の状況を評価する設定になっており，発症から 48 時間経過しないと中等症か重症かを判定することができないという問題点がある [3)].

急性膵炎の中には入院後 48 時間以内の死亡例も存在し，そのような症例では改訂アトランタ分類での重症度判定を行うことができない．入院時に急性膵炎の重症度を迅速に判断することができれば，早期より効率的かつ適切な対処が行われることが期待される．

そこで今回，急性膵炎にて入院加療を行った症例を後方視的に検証することで入院時に測定可能な項目を評価し，死亡危険因子の検討を行うことを計画した．

4. 目的

急性膵炎にて入院加療を行った症例を後方視的に検討し，入院時に評価できる項目における死亡の危険因子を検証する．それによって急性膵炎の重症度を迅速に判断し，効率的かつ適切な対処が行われるようにすること．

5. 研究の方法

5. 1. 対象疾患又は病態

2013 年 4 月より 2017 年 12 月までに，当院および豊川市民病院で入院加療を行った急性膵炎の症例．

5. 2. 対象者の選択基準･除外基準と、対象者の選定方法

（1）選択基準

急性膵炎と診断し，入院加療を行った症例を対象とする．

（2）除外基準

入院時に CT が施行されていない症例，他施設において急性膵炎と診断後に 2 日間以上の加療が行われていた症例，および 15 歳未満の症例は除外対象患者とする．

（3）対象者の選定方法

2013 年 4 月より 2017 年 12 月までに，当院および豊川市民病院で入院加療を行った急性膵炎の全症例を抽出する．

5. 3. 目標症例数

症例数：200 例．

5. 4. 研究の期間

承認日から 2 年間．

5. 5. 研究プロトコール

当院および豊川市民病院において急性膵炎と診断し，入院加療を行った症例において以下の評価項目を検証する.

- 年齢
- 性別
- 急性膵炎の成因
- 転帰
- 発症から来院までの時間
- CT 所見
- 既往歴
- 血液検査所見
- 入院時のバイタルサイン
- BMI

6. 個人情報等の取り扱い

研究責任・分担者および本研究の実施に携わる全ての者は，個人情報保護法に基づき被験者の情報を適正に取り扱い，個人情報を保護する．被験者の個人情報とは診察，検査などにより実施医療機関が知り得る全ての情報の事で，氏名，生年月日，診断名，臨床検査値などをいう.

症例報告その他への被験者の個人情報の記載が必要な場合は，研究責任・分担医師が連結可能匿名化を行い、すべて被験者識別コードを用い，被験者の個人情報が遺漏することのないように配慮する．研究責任者は，カルテ番号・被験者氏名・生年月日と施設にて設定した被験者識別コード対応表を作成する．コード表は研究責任者が原本を保管する.

7. 研究の資金源

本研究は通常診療の後方視的観察研究であり，研究自体に特段の資金源を必要としない.

8. 本研究に伴い発生する利益相反

本研究の計画･実施･発表に関して可能性のある利益相反はない.

9. データの取り扱い

研究責任者は研究の実施に関わる重要な文書（申請書類の控え，医学研究科長･病院長からの通知文書，各種申請書･報告書の控えなど）を研究の中止または終了後 3 年が経過した日までの間保存する.

10. 研究情報の公開

本研究についての情報を臨床研究開発支援センターホームページに公開し，研究が実施又は継続されることについて、研究対象者等が拒否できる機会を保障する.

11. 研究の終了と報告書の提出

研究が終了した場合，研究責任者は，「実施状況報告書（終了）」を医学研究科長･病院長（窓口：臨床試験管理センター）に提出する.

12. 実施計画書の改訂

実施計画書の変更が必要となった場合，研究責任医師は臨床研究審査委員会の承認を得て行う．研究責任・分担医師は，医学的にやむを得ない場合を除き，実施計画書からの逸脱または変更を行ってはならない.

13. 倫理的事項

本研究に関係するすべての研究者は「ヘルシンキ宣言」および｢人を対象とする医学系研究に関する倫理指針｣（文部科学省･厚生労働省告示第 3 号）および実施計画書に従って本研究を実施する.

14. 引用文献

1. Banks PA, Bollen TL, Dervenis C, et al. Classification of acute pancreatitis-2012: revision of the Atlanta classification and definitions by international consensus. Gut. 2013; 62: 102-111
2. Talukdar R, Clemens M, Vege SS. Moderately Severe Acute Pancreatitis: Prospective Validation of This New Subgroup of Acute Pancreatitis. Pancreas. 2012; 41: 306-309
3. Talukdar R, Bhattacharrya A, Rao B, et al. Clinical utility of the Revised Atlanta Classification of acute pancreatitis in a prospective cohort: Have all loose ends been tied? Pancreatology. 2014; 14: 257-262

15. 別添資料

説明同意文書

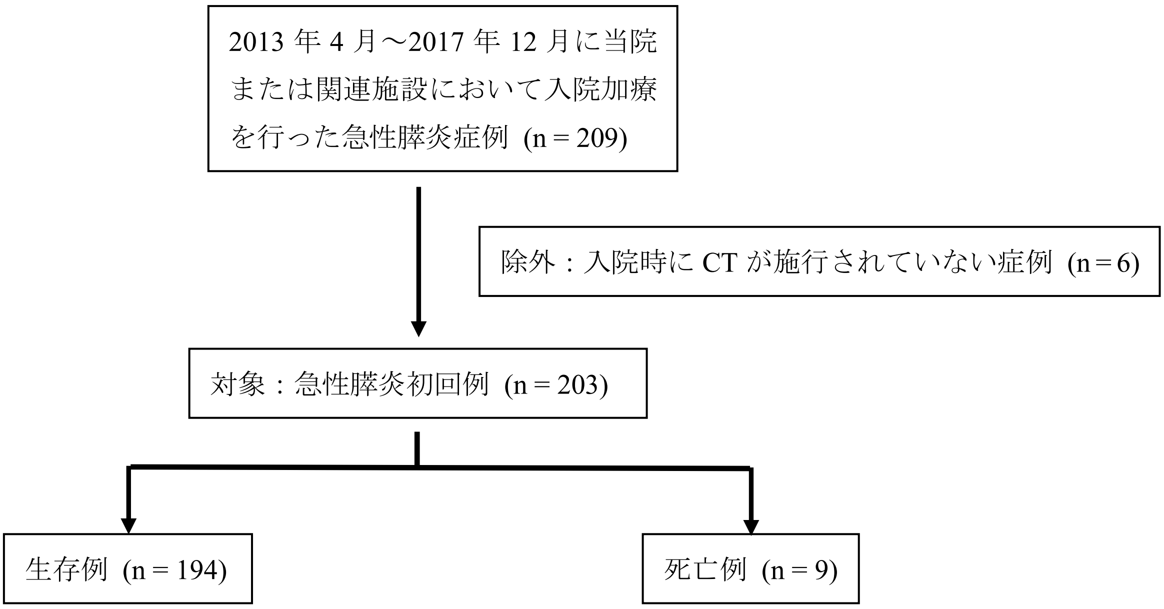

除外基準

・入院時に CT が施行されていない症例

・当院または関連施設以外の施設において急性膵炎と診断され，診断から 2 日間以上経過した後に当院または関連施設へ紹介となった症例

・入院時に 15 歳未満の症例

評価項目

・入院時の年齢

・性別

・急性膵炎の成因

・臨床経過（死亡率，入院日数，臓器不全の有無，被包化壊死（walled-off necrosis; WON）発症の有無）

・腹痛等の急性膵炎として矛盾しない症状の発症から来院までの時間（発症時間は担当医の判断による）

・入院時の CT 所見

・既往歴・併存疾患

・入院時の血液検査所見 (WBC, Hct, Plt, CRP, Amylase, LDH, BUN, Creatinine, Calcium, PaO2, PaCO2, Base excess)

・入院時のバイタルサイン

・入院時の BMI

June 1, 2018

1. Official scientific title of the study

Predictive factors for the mortality of acute pancreatitis on admission

2. Contact information

Public contact
Itaru Naitoh, MD
Nagoya City University Graduate School of Medical Sciences
Department of Gastroenterology and Metabolism
1 Kawasumi, Mizuho-cho, Mizuho-ku Nagoya 467-8601, Japan
inaito@med.nagoya-cu.ac.jp

Research contact person
Naruomi Jinno, MD
Nagoya City University Graduate School of Medical Sciences
Department of Gastroenterology and Metabolism
1 Kawasumi, Mizuho-cho, Mizuho-ku Nagoya 467-8601, Japan
naru1211@med.nagoya-cu.ac.jp

Research Collaborator
Yasuki Hori, MD, Katsuyuki Miyabe, MD, Michihiro Yoshida, MD, Makoto Natsume, MD, Akihisa Kato, MD, Go Asano, MD, Hitoshi Sano MD, and Kazuki Hayashi, MD

Participating institutions
Nagoya City University Graduate School of Medical Sciences and Toyokawa City Hospital

3. Background and study aims

Acute pancreatitis (AP) is a common but heterogeneous pancreatic disease, ranging from mild disease to disease associated with high morbidity and mortality. Despite recent advances in diagnostic and evidence-based therapeutic management, almost 20% of patients with AP develop a complicated clinical course that requires long hospitalization, intensive care, and invasive interventions; furthermore, the condition can result in mortality. The 1992 Atlanta classification was one of the first attempts at grading AP. This classification divides AP into mild and severe groups. The severe group is defined by the presence of organ failure (OF) as well as local and systemic complications. Recently, two new classification systems have been developed: the 2012

revised Atlanta classification nd the determinant-based classification. These two scales introduce the concept of persistent OF as a major determinant for the prognosis of AP. However, based on these classifications, disease severity is determined after 48 hours of admission, but AP patients sometimes die within this timeframe. Therefore, the factors that predict mortality should be further explored to provide timely treatment for AP. The aim of this study was to investigate the predictive factors of mortality in patients with AP on admission.

4. Objectives

To investigate the predictive factors for mortality in patients with acute pancreatitis on admission

5. Methods

Study type
Observational

Study period
From April 1, 2013 to December 31, 2017

Eligibility
Age-lower limit: ≧15 years old
Age-upper limit: N/A
Gender: Male and Female
Key inclusion criteria: Patients who diagnosed as acute pancreatitis
Key exclusion criteria: Lacking CT image on admission
Target sample size: 200

6. Patient information

All patient’s data are anonymized and managed by a person in charge of this study.

7. Funding

N/A

8. Conflict of interest

N/A

9. Handling of the data

All anonymized patient’s information and documents are archived 3 years after the end of the

study.

10. Expose

The study protocol is uploaded (http://ncu-cr.jp/patient), and study participants can refuse to entry this study.

11. Report

After finishing the study, the researcher must report to the Review Board of the Nagoya City University Graduate School of Medical Sciences.

12. Revision

If the researcher would like to change or revise this study protocol, the researcher must contact to the Review Board of the Nagoya City University Graduate School of Medical Sciences.

13. Ethics

The study must accomplish in accordance with the Declaration of Helsinki.

14. References

- Banks PA, Bollen TL, Dervenis C, et al. Classification of acute pancreatitis-2012: revision of the Atlanta classification and definitions by international consensus. Gut. 2013; 62: 102-111
- Talukdar R, Clemens M, Vege SS. Moderately Severe Acute Pancreatitis: Prospective Validation of This New Subgroup of Acute Pancreatitis. Pancreas. 2012; 41: 306-309
- Talukdar R, Bhattacharrya A, Rao B, et al. Clinical utility of the Revised Atlanta Classification of acute pancreatitis in a prospective cohort: Have all loose ends been tied? Pancreatology. 2014; 14: 257-262

15. Annex

Written informed consent form is uploaded (http://ncu-cr.jp/patient).

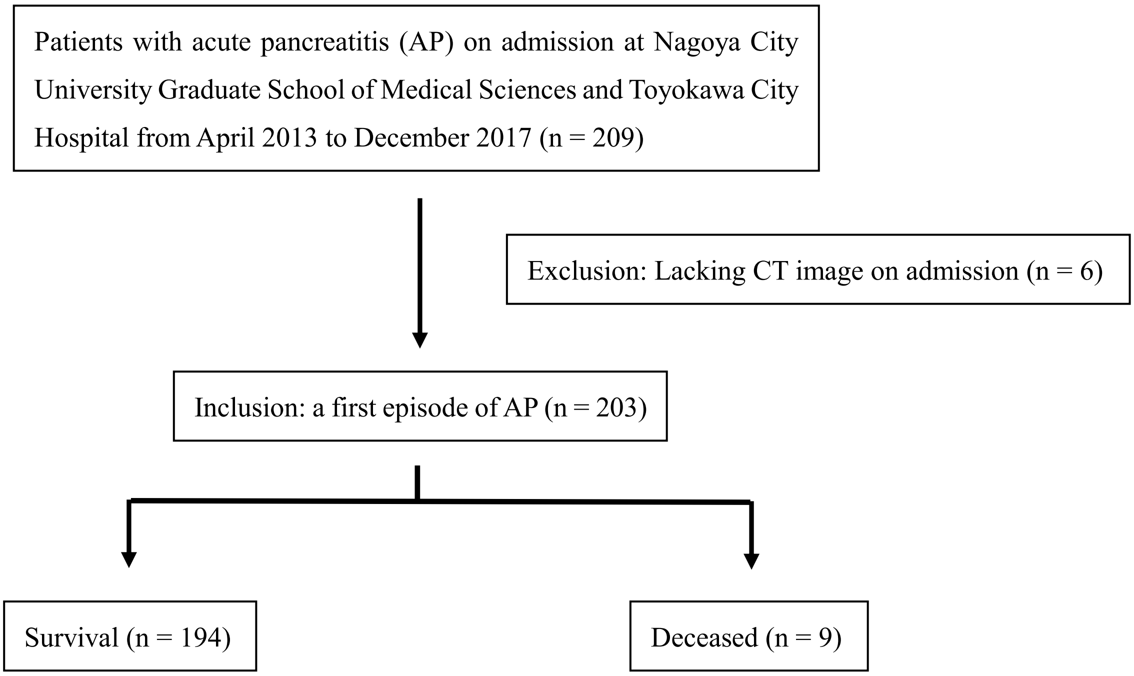

Exclusion criteria

・Lacking CT image on admission
・Under 15 years old

Evaluation items

・Age
・Sex
・Etiology of AP
・Clinical outcomes (mortality, days of hospitalization, organ failure, walled-off necrosis)
・Time to visit hospital from initial symptom
・CT findings
・Past medical history
・Hematology findings (white blood cell, hematocrit, platelets, C-reactive protein, amylase, lactate dehydrogenase, blood urea nitrogen, creatinine, calcium, PaO2, PaCO2, and base excess)
・Vital signs
・Body mass index
