# Supplementary material for: Predictive factors for the mortality of acute pancreatitis on admission
Source: PLoS One. 2019 Aug 22;14(8):e0221468. doi: 10.1371/journal.pone.0221468 (PMC6706052; doi:10.1371/journal.pone.0221468)
Supplement: S3 Fig — (PDF) [file pone.0221468.s003.pdf]

急性膵炎にて入院加療を受けられた方
(2013年5月～2017年12月の期間)を対象にした研究

作成日：2018年7月26日

臨床研究課題名

急性膵炎の入院時における予後予測因子の検討

1．　この研究を計画した背景

急性膵炎とは膵臓の急性炎症で，他の隣接する臓器や遠隔臓器にも影響を及ぼしえる疾患です．発生頻度は5～80/10万人/年であり，近年増加傾向にあります．ガイドラインの普及により急性膵炎診療の標準化が確立されつつあり，急性膵炎の死亡率は7.4%（1995～1998年）から2.6%（2011年）へと改善傾向にあります．軽症急性膵炎は治療にて速やかに軽快する一方，重症急性膵炎は集学的治療を行うにも関わらず，いまだ高い死亡率であり，特に最重症の場合は死亡率43%と高率に死に至る疾患です．

急性膵炎の重症度分類として2012年に改訂アトランタ分類が提唱され，標準的に利用されています．しかしながらそれは，発症から48時間経過した際の臓器不全の評価を用いるため，早期の重症度判定が困難です．

急性膵炎には入院後48時間以内の早期死亡例も存在し，そのような症例では改訂アトランタ分類での重症度判定を行うことができません．より早期（入院時）に重症度を判断することができれば，重症化を視野に入れた早期治療介入が可能となります．今回我々は，当院および関連施設にて入院加療を行った急性膵炎症例を後方視的に検証し，入院時における死亡予測因子の検討することを計画しました．

2．　この研究の目的

急性膵炎にて入院加療を行った症例を後方視的に検証し，入院時における死亡予測因子を検討します．それによってより早期（入院時）に重症度を判断し，重症化を視野に入れた早期治療介入を可能とすることを目的とします．

なお、この研究は，以下研究者によって本院にて実施しています．

研究責任医師：名古屋市立大学病院　消化器代謝内科学　内藤　格

3．　この研究の方法

2013年5月より2017年12月までに，当院および豊川市民病院での入院期間中のあなたの医療記録より以下のようなデータをこの研究に利用させていただきます。

- 入院時の年齢
- 性別
- 急性膵炎の成因

・病状の経過
・発症から来院までの時間
・入院時の CT 検査の所見
・既往歴や合併症
・入院時の採血検査の結果
・入院時の体温、血圧、脈拍など
・入院時の体格指数（BMI：肥満や痩せの目安となるもの）

4．　この研究に参加しなくても不利益を受けることはありません.

この臨床研究への参加はあなたの自由意思によるものです．この臨床研究に同意された後であっても，今回追加された解析にあなたの臨床情報を使用することについて，いつでも参加を取りやめることができます．途中で参加をとりやめる場合でも，今後の治療で決して不利益を受けることはありません.

5．　あなたのプライバシーに係わる内容は保護されます.

試験を通じて得られたあなたに係わる記録が学術雑誌や学会で発表されることがあります．しかしあなたの情報は匿名化した番号で管理されるため，得られたデータが報告書などであなたのデータであると特定されることはありませんので，あなたのプライバシーに係わる情報（住所・氏名・電話番号など）は保護されます.

6．　得られた医学情報の権利および利益相反について

本研究により予想される利害の衝突はないと考えています．本研究に関わる研究者は「厚生労働科学研究における利益相反（Conflict of Interest：COI）の管理に関する指針」を遵守し，各施設の規定に従って COI を管理しています.

7．　この研究は必要な手続きを経て実施しています。

この研究は，公立大学法人　名古屋市立大学大学院　医学研究科長および名古屋市立大学病院長が設置する医学系研究倫理審査委員会（所在地：名古屋市瑞穂区瑞穂町字川澄１）において医学，歯学，薬学その他の医療又は臨床試験に関する専門家や専門以外の方々により倫理性や科学性が十分であるかどうかの審査を受け，実施することが承認されています．またこの委員会では，この試験が適正に実施されているか継続して審査を行います。

なお，本委員会にかかわる規程等は，以下、ホームページよりご確認いただくことができます.

名古屋市立大学病院　臨床研究開発支援センター　ホームページ　“患者の皆様へ”
http://ncu-cr.jp/patient

8．　本研究について詳しい情報が欲しい場合の連絡先

この臨床研究について知りたいことや，ご心配なことがありましたら，遠慮なくご相談ください．また，この研究にあなたご自身のデータを使用されることを希望されない方は，ご連絡ください.

名古屋市立大学病院　臨床研究開発支援センター
連絡先　平日（月～金）　8:30～17:00　TEL(052)858-7215

July 26, 2018

1. Title of the study

Predictive factors for the mortality of acute pancreatitis on admission

2. Background and study aims

Acute pancreatitis (AP) is a common but heterogeneous pancreatic disease (5-80 cases / 100,000 persons / year), ranging from mild disease to disease associated with high morbidity and mortality. In recent years, treatment of AP has been progressed, and the mortality rate become lower compared with the past (from 7.4% [1995-1998] to 2.6% [2011]). Despite recent advances in diagnostic and evidence-based therapeutic management, severe AP develop a complicated clinical course that requires long hospitalization, intensive care, and invasive interventions; furthermore, the condition can result in mortality.

The 1992 Atlanta classification was one of the first attempts at grading AP. This classification divides AP into mild and severe groups. The severe group is defined by the presence of organ failure (OF) as well as local and systemic complications. However, based on the classification, disease severity is determined after 48 hours of admission, but AP patients sometimes decease within this timeframe. Therefore, the factors that predict mortality on admission should be explored to provide the adequate treatment for AP. The aim of this study was to investigate the predictive factors of mortality in patients with AP on admission.

3. Methods

Study type: observational and retrospective design

Study period: from April 1, 2013 to December 31, 2017

Evaluation items

・age
・sex
・the etiology of AP
・clinical outcomes (mortality, hospitalization, OF, walled-off necrosis)
・time to hospital visit from initial symptom onset
・CT findings
・past medical history
・hematology findings
・vital signs
・body mass index

4. You don’t receive any disadvantage even if you don’t entry this study.

You have a right to decline your participation anytime.

5. Your privacy is protected.

All patients' data are anonymized and managed by a person in charge of this study.

6. Conflict of interest

No potential conflict of interest relevant to this study.

7. This study was approved by Institutional Review Board of Nagoya City University Hospital.

This study was approved by an Institutional Review Board and the study protocol is uploaded (http://ncu-cr.jp/patient).

8. Contact information

Clinical research management center in Nagoya City University Hospital

Phone: +81-52-858-7215
